# Barriers to healthcare access and experiences of stigma: Findings from a coproduced Long Covid case‐finding study

**DOI:** 10.1111/hex.14037

**Published:** 2024-04-18

**Authors:** Donna Clutterbuck, Mel Ramasawmy, Marija Pantelic, Jasmine Hayer, Fauzia Begum, Mark Faghy, Nayab Nasir, Barry Causer, Melissa Heightman, Gail Allsopp, Dan Wootton, M. Asad Khan, Claire Hastie, Monique Jackson, Clare Rayner, Darren Brown, Emily Parrett, Geraint Jones, Rowan Clarke, Sammie Mcfarland, Mark Gabbay, Amitava Banerjee, Nisreen A. Alwan, The STIMULATE‐ICP Consortium

**Affiliations:** ^1^ School of Primary Care Population Sciences and Medical Education University of Southampton Southampton UK; ^2^ Institute of Health Informatics University College London London UK; ^3^ Brighton and Sussex Medical School University of Sussex Falmer UK; ^4^ Department of Social Policy and Intervention University of Oxford Oxford UK; ^5^ PPIE Co‐applicant for STIMULATE‐ICP London UK; ^6^ Member of the Community Advisory Board as person with lived experience of Long Covid Southampton UK; ^7^ University Hospitals of Derby and Burton NHS Foundation Trust Derbyshire UK; ^8^ Clinical Exercise and Rehabilitation Research Centre University of Derby Derby UK; ^9^ Department of Health and Social Care Office for Health Improvement and Disparities UK; ^10^ Merton Public Health, Merton Council London UK; ^11^ University College London Hospitals NHS Trust London UK; ^12^ Royal College of General Practitioners London UK; ^13^ Clinical Infection Microbiology and Immunology University of Liverpool Liverpool UK; ^14^ Liverpool University Hospitals NHS Foundation Trust Liverpool UK; ^15^ Manchester University Hospitals NHS Foundation Trust Manchester UK; ^16^ Long Covid Support Charity London UK; ^17^ Chelsea and Westminster Hospital NHS Foundation Trust London UK; ^18^ Long Covid Kids Charity Salisbury UK; ^19^ NIHR Applied Research Collaboration North West Coast Liverpool UK; ^20^ Department of Primary Care and Mental Health University of Liverpool Liverpool UK; ^21^ University Hospital Southampton NHS Foundation Trust Southampton UK; ^22^ NIHR Applied Research Collaboration Wessex Southampton UK

**Keywords:** epistemic injustice, health inequalities, Long Covid, stigma

## Abstract

**Background and Aim:**

Long Covid is often stigmatised, particularly in people who are disadvantaged within society. This may prevent them from seeking help and could lead to widening health inequalities. This coproduced study with a Community Advisory Board (CAB) of people with Long Covid aimed to understand healthcare and wider barriers and stigma experienced by people with probable Long Covid.

**Methods:**

An active case finding approach was employed to find adults with probable, but not yet clinically diagnosed, Long Covid in two localities in London (Camden and Merton) and Derbyshire, England. Interviews explored the barriers to care and the stigma faced by participants and were analysed thematically. This study forms part of the STIMULATE‐ICP Collaboration.

**Findings:**

Twenty‐three interviews were completed. Participants reported limited awareness of what Long Covid is and the available pathways to management. There was considerable self‐doubt among participants, sometimes reinforced by interactions with healthcare professionals (HCPs). Participants questioned their deservedness in seeking healthcare support for their symptoms. Hesitancy to engage with healthcare services was motivated by fear of needing more investigation and concerns regarding judgement about the ability to carry out caregiving responsibilities. It was also motivated by the complexity of the clinical presentation and fear of all symptoms being attributed to poor mental health. Participants also reported trying to avoid overburdening the health system. These difficulties were compounded by experiences of stigma and discrimination. The emerging themes reaffirmed a framework of epistemic injustice in relation to Long Covid, where creating, interpreting and conveying knowledge has varied credibility based on the teller's identity characteristics and/or the level of their interpretive resources.

**Conclusion:**

We have codeveloped recommendations based on the findings. These include early signposting to services, dedicating protected time to listening to people with Long Covid, providing a holistic approach in care pathways, and working to mitigate stigma. Regardless of the diagnosis, people experiencing new symptoms must be encouraged to seek timely medical help. Clear public health messaging is needed among communities already disadvantaged by epistemic injustice to raise awareness of Long Covid, and to share stories that encourage seeking care and to illustrate the adverse effects of stigma.

**Patient or Public Contribution:**

This study was coproduced with a CAB made up of 23 members including HCPs, people with lived experience of Long Covid and other stakeholders.

## 1 BACKGROUND

Long Covid is the patient‐coined term for prolonged ill health following acute infection with severe acute respiratory syndrome coronavirus 2 (SARS CoV‐2).^1^, ^2^ Postcoronavirus disease 2019 (COVID‐19) condition (Long Covid) occurs after ‘probable or confirmed SARS CoV‐2 infection, usually 3 months from the onset of COVID‐19 with symptoms and that last at least 2 months and cannot be explained by an alternative diagnosis’.^1^ In March 2023, the Office for National Statistics estimated that 1.9 million people were living with Long Covid in the United Kingdom.^3^

Long Covid can cause varying symptoms and impact on daily life, including, but not limited to, fatigue, breathlessness, headache, cognitive dysfunction, chest pain, muscle/joint pains, cough, disturbed sleep and neuropsychiatric symptoms.^1^, ^4^, ^5^, ^6^, ^7^, ^8^ It can have wide‐ranging negative social impacts that include inhibiting the ability to engage in employment and creating financial burdens.^4^, ^9^, ^10^, ^11^

Health inequalities exist between different population groups in England.^12^, ^13^ Inequalities were evident before COVID‐19 but the pandemic has exacerbated them and increased their visibility.^12^ COVID‐19 resulted in higher infection and mortality rates for ethnic minorities and people living in more deprived areas.^14^, ^15^ Little is known about disadvantaged groups' experiences of Long Covid. Some evidence suggests that people living in more deprived communities and who belong to some ethnic minority backgrounds are more likely to experience Long Covid than those living in the least deprived areas or those of White ethnicity^16^, ^17^, ^18^ but conversely may be underrepresented in post‐COVID services.^19^ This could suggest fewer people with Long Covid symptoms from these disadvantaged groups are receiving Long Covid care, which has the potential to increase health inequalities.

Emerging evidence suggests that Long Covid is often stigmatised.^20^, ^21^ Analysis of survey data of almost 900 people living with Long Covid in the United Kingdom found widespread stigmas experienced by people with Long Covid.^20^ In this study, the Long Covid Stigma Scale (LCSS) measured three recognised mechanisms of stigma. These included direct experiences of discrimination (enacted stigma), expectations of poor treatment (anticipated stigma) and self‐stereotyping based on negative connotations of having the condition (internalised stigma).^22^, ^23^, ^24^, ^25^

Stigma can occur by not conforming to standards that society calls normal.^26^ Stigmatised people are thus ‘disqualified from full social acceptance’ due to an attribute deemed to be ‘deeply discrediting’.^26^ A wide range of long‐term conditions (LTCs) can be considered stigmatising^27^ and stigma is known to adversely affect help‐seeking, as well as physical and mental health outcomes.^28^ Although anyone can experience stigma, its effects can be amplified for socioeconomically disadvantaged and minority groups.^28^

Experiences and expectations of discrimination can lead to poor engagement with healthcare services.^12^ Choices about revealing illness status are often bound up with expected stigmatising consequences for people with chronic conditions.^29^ Mental health stigma^28^, ^30^ and weight‐based prejudice^28^, ^31^ can prevent help‐seeking and the intersection with ethnicity, culture and gender can increase its impact.^28^ Additionally, experiences or feelings of social exclusion are not uncommon in health stigma literature.^28^ Similarly, internalised stigma, including feelings of shame or self‐comparisons with others, has been associated with chronic conditions.^29^

The experiences of people living with Long Covid can create epistemic injustice as suggested by Ireson et al.^32^ In their analysis of online patient stories, they found that people with Long Covid are frequently disbelieved with their testimonies often not taken seriously. Epistemic injustice is inequality surrounding knowledge^33^ and can occur as ‘hermeneutical injustice’ (where an individual does not have the means to interpret their ‘experiences’) and ‘testimonial injustice’ (discriminatory mistrust of an individual's knowledge).^33^ Both are relevant in the case of Long Covid. It is, therefore important to consider how multiple dimensions of disadvantage intersect when exploring health stigma and other barriers to care.

We aimed to coproduce a community‐based approach for identifying people with probable Long Covid, who have not yet received a formal clinical diagnosis, for the purpose of understanding the barriers and stigma they face when attempting to access support and care.

## 2 METHODS

This work forms part of the National Institute for Health and Care Research Symptoms, Trajectory, Inequalities and Management: Understanding long COVID to Address and Transform Existing Integrated Care Pathways (STIMULATE‐ICP) study. Methods were detailed in a published protocol.^34^

### 2.1 Coproduction with a Community Advisory Board (CAB)

Research coproduction is where researchers work with public contributors, including patients, to design and/or conduct research.^35^, ^36^ A key benefit is generating knowledge that is more meaningful to the population group being studied.^36^, ^37^ In this study, a CAB was formed. This was made up of 10 healthcare professionals (HCPs) (including seven who are also living with Long Covid), eight people living with, or caring for children with, Long Covid and five other stakeholders from voluntary organisations and local public health bodies.

Six CAB meetings took place between February 2022 and July 2023. Discussions in these meetings centred around defining and refining study aims and methodology, planning the implementation of study findings and dissemination. The CAB also contributed to the design, review and production of study documents and members are coauthors on this paper.

### 2.2 Study design

The purpose of using an active case finding approach was to find individual cases of probable Long Covid that have not been clinically diagnosed. This study was initially conducted in the London Borough of Camden, England. Two other sites later joined the study: the London Borough of Merton and the county of Derbyshire, England. Health inequalities exist within each of these localities.^38^, ^39^, ^40^ Camden is the most ethnically diverse area—41% of people residing in Camden come from an ethnic minority background.^38^ An estimated 37% of Merton residents are from an ethnic minority group and inequalities exist between the east and west of the borough.^39^, ^41^ In contrast to the London sites, a large part of Derbyshire is rural and 6.3% of the population are from an ethnic minority background.^42^

**Figure 1:**
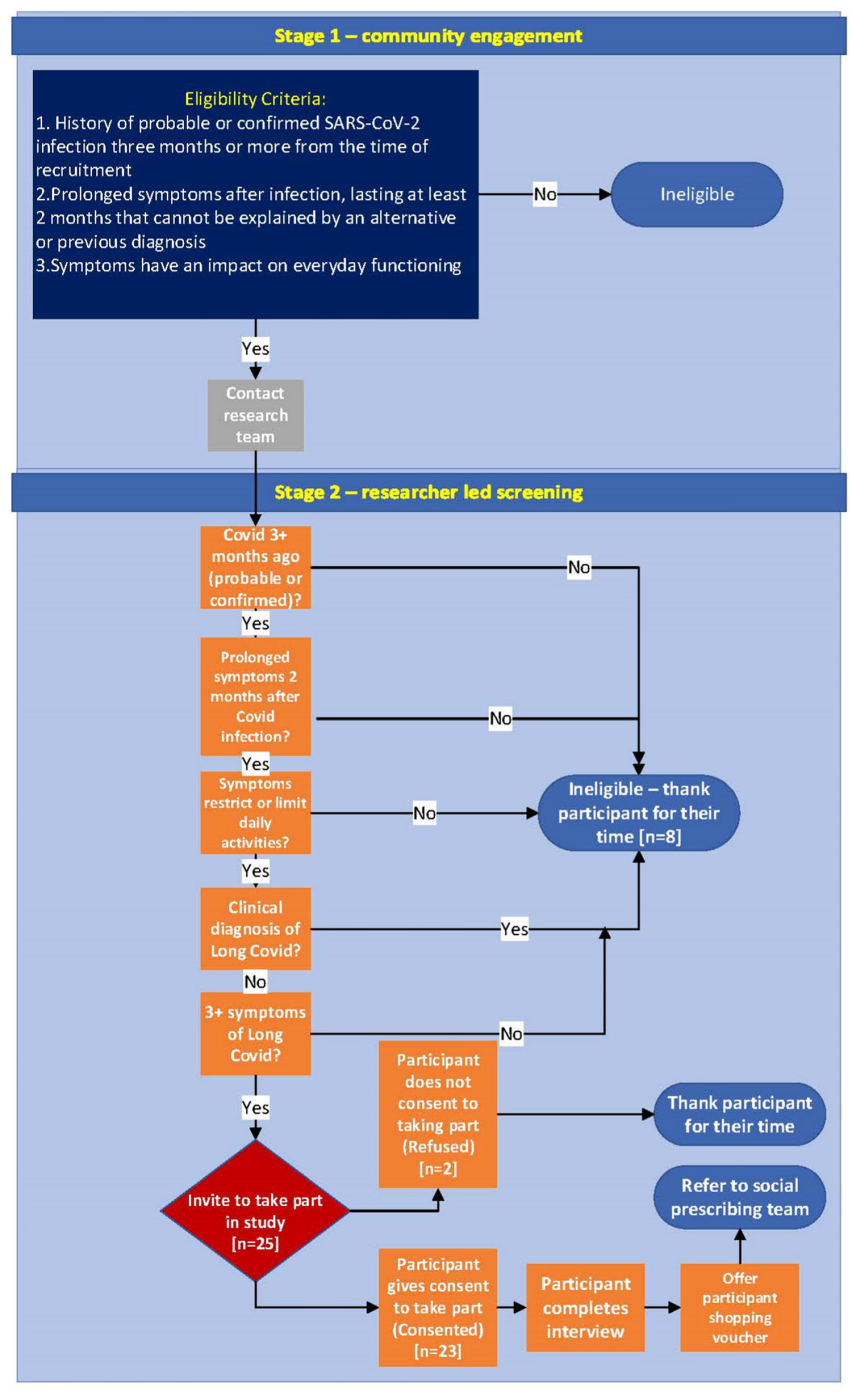
Study eligibility flow chart.

A leaflet raising awareness of the study and some of the most common symptoms of Long Covid was disseminated within communities in the participating localities, with the aid of community organisations, local authorities and social prescribing teams.^34^ Sharing study materials via voluntary organisations was the most fruitful way to access communities in this study. Individuals who met the eligibility criteria were assumed to have probable Long Covid and were invited to take part in a qualitative interview. The eligibility criteria were agreed with the CAB (Figure 1, Supporting Information S1: Materials A and B) and are detailed in the study protocol.^34^

### 2.3 Qualitative research data collection

Semi‐structured interviews were completed with people with probable Long Covid across Camden, Merton and Derbyshire. The interview topic guide was coproduced with the CAB. This guide consisted of predominantly open questions investigating barriers to obtaining care for Long Covid symptoms. It also included some questions from the LCSS^20^ exploring possible stigmatising experiences across the three mechanisms of enacted (overt experiences of being treated unfairly), anticipated (expectation of being treated unfairly) and internalised (adopting negative stereotyping and applying to self) stigma. Prompts were used to find out the context of these experiences, and to provide an in‐depth exploration of the three stigma mechanisms.

All interviews were conducted by D. C. between August 2022 and May 2023. Most completed by videocall, four were completed via a telephone call. Informed consent was obtained from participants before undertaking the interviews. Participants were reminded at the beginning of the interviews that study eligibility did not constitute a clinical diagnosis of Long Covid. Upon completion of the interview, participants were offered a shopping voucher as a thank you gesture, and a referral to a social prescribing service to receive personalised support. Social prescribing aims to improve mental and physical health through referral to community‐based services for holistic support.^43^ Participants were also offered a letter indicating study participation that could be taken to their primary care team. A full outline of the methods is in the study protocol.^34^

### 2.4 Analysis

Thematic analysis, based on the approach described by Braun and Clarke,^44^, ^45^ was employed to analyse the interviews. After becoming familiar with the transcripts, D. C. completed initial coding using predominantly an inductive approach using NVivo.^46^ Some codes matched the concepts studied in the LCSS so more deductive coding was applied in relation to these questions. M. R. completed additional coding on a subsample of transcripts to ensure intercoder reliability. Once coding had been completed D. C., M. R. and N. A. A. met multiple times to discuss potential themes. The LCSS informed the creation of themes related to experiences of enacted, anticipated and internalised stigma.^20^ A thematic framework was developed in light of the findings and agreed upon before drafting this paper.

## 3 RESULTS

Twenty‐three semi‐structured interviews were completed with participants who reported varying symptoms consistent with Long Covid, but no known clinical diagnosis. Information regarding these symptoms can be found in Supporting Information S1: Material C. Participants' characteristics can be found in Table 1. Most participants were from the Camden locality.

**Table 1:** Participant characteristics.

Gender	
Female	16
Male	7
Ethnicity	
White British	8
White/White other	6
Asian/Asian British	5
Black/Black African/Black Caribbean/Black British	2
Mixed/multiple ethnic groups/other	2
Age	
30–39	5
40–49	5
50–59	6
60–69	4
70–79	3
Index of multiple deprivation quintile^a^	
1 = Most deprived areas	4
2	5
3	7
4	3
5 = Least deprived areas	3
Unknown	1
Disability status	
Disabled	6
Not disabled	17
Long term conditions present before COVID‐19	
Yes	19
No	4

Abbreviation: COVID‐19, coronavirus disease 2019.

^a^ Index of multiple deprivation which measures areas of deprivation in localities within England. English indices of deprivation 2019. Available from https://www.gov.uk/government/statistics/english-indices-of-deprivation-2019.

A minority were unsure about their Long Covid diagnosis status. It was assumed that these participants did not have a formal diagnosis and so they were included in this study. This was on the basis that some of these participants noted Long Covid had been suggested as a possibility by HCPs, but they had had no follow‐up investigations. Two of the participants believed referrals might have been made but these had not yet been fulfilled.

Four key themes were identified during analysis: (1) Uncertainty and Long Covid awareness, (2) Barriers to engagement with the healthcare system, (3) Experiences and perceptions of stigma and discrimination and (4) Sources of support. The following sections focus on these themes.

### 3.1 Theme 1: Uncertainty and Long Covid awareness

#### 3.1.1 Awareness of Long Covid and Long Covid support services

A lack of adequate awareness of Long Covid was prominent in the accounts of participants. Some participants were not aware of the existence of Long Covid as a health condition, others had some awareness but did not equate their symptoms to Long Covid.

My symptoms as such have never really gone away but I've not always attributed them to Long Covid or whatever. (Female, 60–69, White British)

Participants were more likely to describe not feeling ‘normal’ or not feeling ‘the same since’ their COVID‐19 infection. Others tended to connect Long Covid with more ‘severe’ symptoms. Two participants only became aware of Long Covid upon engagement with the study information.

I wasn't aware of it, but now you've kind of made me aware. (Female, 30–39, Bangladeshi–British)

Other participants suggested that the study information reinforced their belief that their symptoms were Long Covid.

Some participants assumed lack of knowledge and awareness of Long Covid within the National Health Service (NHS).

They still don't know what to do and they don't know what to say. (Female, 70–79, White British)

There was a lack of awareness of how to obtain care for symptoms and some felt there was no support available.

I don't think there's any cure. There's nothing they can do. (Female, 70–79, Jewish)

Furthermore, some participants indicated that they would seek help if they felt that treatment was available. A need for increased awareness, both within the community and in healthcare, was stressed by some.

Awareness is so important to even get a diagnosis or think, well, it could potentially be that, and it's the same with professionals. (Female, 30–39, Bengali)

#### 3.1.2 Self‐doubt

Self‐doubt prevented some people with Long Covid symptoms from seeking help. Participants were sometimes unsure if their symptoms were caused by Long Covid, and some explained their symptoms as potentially resulting from ageing, menopause or stress.

Because I've had just a lot of life changes, you know, like in terms of my career, what's going on with work, with stress, all of that, I've then as a result not really felt that comfortable saying this might be something else as well [laughing]. (Male, 30–39, Black British Caribbean)

There was also evidence of participants downplaying symptoms or comparing their experiences to others.

I didn't really think it would be right to say it was Long Covid is having read about people suffering from Long Covid as having really, really severe symptoms and not being able to do anything and thinking this doesn't apply to me. (Male, 60–69, Asian British)

Sometimes, interactions with HCPs reinforced this self‐doubt. For example, the gendered experience of women who suspected their symptoms could be menopause‐related had this confirmed by GPs without further investigation.

I kind of spoke to a doctor about the fatigue. And the dizziness and stuff. And he just went, oh, yes, it just sounds like perimenopause, just got to get on with it and that was kind of it. So, that was as far as it went because I thought I don't really want to bother them again. (Female, 40–49, White)

Likewise, if Long Covid was not suspected by HCPs, some participants rejected it as a possibility.

So I started Googling it, and then I thought, This sounds like what I have. But because the GPs didn't mention it, I just thought, they're the professionals. They know me best in terms of medically, and they know my medical history. So I was leaning towards oh, it can't be. (Female, 30–39, Bengali)

Participants trusted HCPs.

You think you're talking to a consultant and health professionals that they know better. (Female, 50–59, White)

Participants lacked the knowledge and awareness needed to interpret and describe their symptoms as being attributable to Long Covid and sometimes experienced self‐doubt surrounding their cause. This worked in tandem with the (perceived) lack of knowledge among, and privileged perspectives of, HCPs to avert participant help‐seeking. These experiences equate to epistemic injustice.

### 3.2 Theme 2: Barriers to engagement with healthcare

#### 3.2.1 Hesitancy around seeking support from the healthcare system

Hesitancy around seeking treatment or care was apparent for different reasons. One participant was concerned that if his symptoms were not associated with Long Covid, further investigation might be needed.

There's a possibility that it's not Long Covid then I'd have to … Like that would involve working out what is going on. (Male, 30–39, Black British Caribbean)

Similarly, another participant expressed that he did not want to be prescribed medication and this was preventing him from reporting symptoms to a HCP.

Another participant explained that she was worried about engaging with healthcare in case assumptions about her ability to fulfil her parenting responsibilities were made.

Sometimes I am afraid if I say to them that, you know, they feel that I am not capable to look after my son and my kids, you know. It's kind of a worry behind my head. I wouldn't like to mention it. (Female, 40–49, Bangladeshi)

Again, this is a gendered experience unique to women. More experiences of gender discrimination are explored in theme 3 below.

Reluctance to engage was sometimes tied to not wanting to burden the NHS.

At the moment, I'd probably feel a bit like I was wasting their time … Because I just feel like at the moment the NHS is in a bit of a state. (Male, 30–39, White)

Participants felt that their symptoms were not a priority to a health system which they considered to be overloaded.

Additionally, engaging with healthcare was considered a part of a nonhelp‐seeking identity for some participants. Some participants expressed how they would have to be exceptionally unwell to consider reaching out.

I'm not that kind of, I don't think I can even knock someone's door to ask for help. (Female, 50–59, mixed race)

Hesitancy in accessing care was felt to be exacerbated by perceived problems in primary care, such as long waiting times for appointments, limited appointment times, only being able to focus on one symptom during consultations or waiting for secondary care referrals for individual symptoms to be fulfilled before any further steps are taken.

#### 3.2.2 Nature of multisystem symptoms makes accessing care difficult

For some, the very nature of Long Covid symptoms made accessing treatment or care difficult. One participant was hesitant to seek care from her general practitioner (GP) because of attribution of new symptoms to pre‐existing health problems.

They tend to always assume that my symptoms are consistent with my chronic long‐term health problem. (Female, 50–59, White British)

The fluctuating and relapsing nature of symptoms, which can occur with Long Covid,^1^, ^4^, ^5^, ^6^ acted as a barrier to obtaining care.

Just when you think, oh, they've gone, up they pop. (Female, 50–59, White British)

Sometimes this meant that symptoms were not present at the time of the healthcare consultations or would disappear soon after. Thus, the changing nature of symptoms can mean that patients do not return for follow‐up care.

Sometimes one symptom was more prominent than other experienced symptoms. Unsurprisingly, these participants sought care for the symptom impacting them the most, which resulted in other symptoms being overlooked by the participant and HCPs. This can result in the pattern of symptoms of Long Covid being missed.

One participant had a flare‐up of her existing health condition after an acute infection with COVID‐19, despite this being stable for a long period of time, and so her other symptoms were neglected:

I've seen my GP, but that's been it to be honest. I think once all of my stomach issues kind of kicked in and, you know, it was a bit of a kind of panic [laughs] because you know it can be kind of quite hard and fast when it comes on and so the focus really, probably went on to that to be honest. (Female, 40–49, White British)

Some participants had experiences of symptoms being attributed to mental health conditions by HCPs including one who was offered antidepressant medication. She acknowledged that she had been experiencing low mood, but this was because of her concerns around not being listened to.

I feel upset because he tried to give me Prozac for it, and I'm trying to explain, it's not a Prozac situation here. (Female, 50–59, White British)

Concerns surrounding the consequences of engaging with healthcare services and the complex nature of Long Covid symptoms served as barriers to help‐seeking for participants. These were sometimes related to expectations of discrimination resulting from participant characteristics. This also fits within the framework of epistemic injustice.

### 3.3 Theme 3: Experiences and perceptions of stigma and discrimination

#### 3.3.1 Experiences of discrimination

Experiences of discrimination, both within healthcare and in the community, were evident from the interviews. A layering of disadvantage was experienced by those with multiple stigmatised identities (e.g., older age and female gender).

A few participants described ageism.

I always feel that your age is put against you. (Female, 50–59, White)

Gender discrimination was implicated in the interviews of some female participants, as noted in themes 1 and 2 above. This was also in the form of GPs lacking knowledge of how to treat women, symptoms being more likely to be attributed to mental health and feeling belittled by HCPs, as one participant described in her interaction with a male consultant:

I did find he was very condescending and patronising and, in my view, I felt that was because I was a woman. (Female, 40–49, White British)

Symptoms and participant knowledge of their own bodies were also reported to be dismissed by HCPs.

He literally dismissed it, that was it. And I think that was the problem with the doctors, they just dismiss things sometimes if they don't want to deal with it … There was no, why is this happening. What can we look in to, you know, is it COVID, is it this. There was nothing. (Female, 40–49, White)

Feelings surrounding a lack of understanding and support were prominent among the participants. A few talked about concerns from others regarding transmissibility, not experiencing empathy or sympathy, not being included in social activities, being judged, being ignored, being perceived as ‘lazy’, or receiving unsolicited suggestions for symptom relief which sometimes carried undertones of blame for not doing enough to keep well.

One participant revealed how she only socialised with people with LTCs because of the lack of understanding from others without chronic illness.

My contacts tend to have come with other people who are ill because it's very difficult to have social interaction with people who aren't ill because they understandably lose patience if you can't physically meet up with them. (Female, 50–59, White British)

Additionally, a few participants talked about a lack of understanding and support from employers and colleagues. This was evident in comments around inefficiency or questioning symptoms and sick leave:

My friend and colleague said, ‘Oh, you are trying to make excuses to take annual leave’. …and it made me feel a bit like, you know, upset. And I said to the person, ‘You know, I wasn't making it up. I was unable to get out of bed and I was really struggling to breath[e] some days’. (Female, 40–49, Bangladeshi)

#### 3.3.2 Anticipated stigma

Some participants expected discrimination to occur because of their Long Covid symptoms. Sometimes, this was due to past stigmatising experiences. For some, having a history of being dismissed before Long Covid impacted their view of help‐seeking. For one participant this dismissal had previously occurred due to her weight.

it's almost as if they bring the weight to the focus and then I feel guilty for taking up time. (Female, 60–69, White British)

One participant expressed worries surrounding engagement without proof of his symptoms.

Unless there's something concrete that I could say, look this is, there is a physical symptom, I'm not making it up, I don't really want to engage [laughing] … I do worry that if it is dismissed that then … they could put a little thing down that I'm a bit of a hypochondriac. So I'd rather not bother. (Male, 30–39, Black British Caribbean)

Similarly, others did not want to be labelled an ‘idiot’, receive a ‘quizzical look’, or be considered ‘awkward’. This participant also described how he previously contracted Monkeypox, sought support and was ‘rebuffed a few times’, despite experiencing symptoms and identifying as being in an at‐risk demographic, before being given a positive diagnosis weeks later. For these participants, their avoidance of dismissal was preventing them from seeking care for their symptoms.

Disbelief was seen as resulting from a lack of understanding or due to varying symptoms. Some participants felt that disbelief extended beyond the community into the medical profession. One participant asked the interviewer ‘how accepting is the healthcare profession that Long Covid is real?’ (Female, 60–69, White British), suggesting that she was unsure if Long Covid is taken seriously by HCPs.

A few participants had interactions with others who expressed disbelief, but mostly this was a perception. Some participants saw dismissal as proof of disbelief.

Participants feared judgement from others due to the impact of symptoms or other people's presumptions of the impact of symptoms.

They will presume that … you're not on the ball. (Female, 60–69, White British)

This fear of judgement was a common reason for participants not talking to others about their symptoms. One participant, early on during her interview, stated that she did not feel comfortable talking about mental health with her GP, highlighting the intersectionality of religious background with mental health stigma

I don't feel that comfortable to say everything about it [mental health] and feel the questions sometimes the doctor asks like I would not be very happy. Actually not that comfortable. Maybe I am from a community with religion and some of the [answers] I am not that comfortable to share. (Female, 40–49, Bangladeshi)

Later, she suggested that she would be apprehensive about sharing details of her symptoms with a potential partner as they may not want to marry someone with a LTC. For some participants, the interview was the first time they had talked about their symptoms and/or the possibility of symptoms being related to Long Covid.

Employment worries were evident throughout the interviews. One participant had to switch from a physically demanding role to a ‘desk job’ because of his symptoms. Participants often felt they were not performing as well at work as they did before.

‘A couple of times I've had to say I need to go home because of this … I feel like I'm taking the Mick [not taking work seriously] myself’. (Male, 50–59, Asian)

Another participant (Male, 60–69, White) suggested that if he was looking for a new role, he ‘might keep it [Long Covid] to [himself]’ to avoid negative views preventing him from finding employment.

Another suggested that as Long Covid is not considered a disability, you would not have employment protections.

It's not considered a disability … you could just be fired just like, you're not performing. And you go, well, I've got Long Covid. They go … we're not legally obliged to consider that so, tough. (Male, 30–39, White)

This perhaps goes some way in explaining the worry surrounding employment performance.

#### 3.3.3 Internalised stigma

There were indications of shame from participants about their illness and associated symptoms. When asked if they felt embarrassed about their illness or physical limitations, participant answers focused on symptoms and not performing as well at work or socially. A few participants felt embarrassed as they were not as fit, ‘motivated’ or ‘physically strong’ as they felt they should be.

However, some were embarrassed by illness in general. One participant explained this as resulting from confusion surrounding being ‘infectious’ and another felt that their symptoms were so ‘vague’ it could lead to disbelief from others. One participant suggested that if others know you are ill, ‘it changes their perception of you’ (Female, 40–49, White British).

Symptoms and not being able to do the same things as before were the main reasons why participants felt of less value due to Long Covid.

It kind of affects me, like across the board with the tiredness. And the forgetfulness and stuff. You just feel like a lesser person. (Female, 40–49, White)

Participants stated they felt different to others because of their symptoms, and some participants compared themselves to others who seemed to recover from COVID‐19 quickly.

When asked if anything has happened to make them feel embarrassed, tainted or different, some participants stated that this was an individual thing and that others have not contributed towards these feelings: ‘No, I just think it—Again it's a personal thing that I feel sometimes’ (Female, 40–49, White British).

Experiences of discrimination, as well as anticipated and internalised stigma worked to prevent engagement with healthcare services. These experiences highlight the need to mitigate stigma to promote help‐seeking behaviour.

### 3.4 Theme 4: Sources of support

#### 3.4.1 Sources of treatment outside of the NHS

Just under half of the participants accessed treatment, care and support outside of the NHS. This included using alternative therapies and remedies, experimenting with vitamins and supplements, consulting with family and friends who are trained HCPs, resorting to private healthcare, and making behavioural changes through diet and exercise. One participant asked a HCP during a secondary care appointment to prescribe her medication after her GP initially refused (Supporting Information S1: Material D, Quote 1). Some participants felt they must be creative when seeking out care and support as they perceive mainstream healthcare to be inadequate.

#### 3.4.2 Social and community support

Social and community support such as peer support, gym/physical and outdoor activity groups, as well as pharmacies for advice‐giving were considered useful for people living with Long Covid. Three participants used social media Long Covid support groups to seek advice regarding their symptoms. Additionally, most participants were receptive to the social prescribing referral offered by the study. Support that included social interaction seemed most popular amongst participants (Supporting Information S1: Material D, Quote 2).

Participants described sharing their experiences within their social circles because of their relatable experiences (Supporting Information S1: Material D, Quotes 3 and 4). One revealed that she had shared the study information with her sisters who had also experienced Long Covid symptoms.

Financial support was mentioned in relation to recouping funds that had been spent on alternative therapies or supplements, and for those who are unable to work fulltime. Similarly, employment support was considered beneficial for people living with Long Covid. Some considered hybrid working and working flexibly advantageous to them when suffering Long Covid symptoms.

#### 3.4.3 Desired healthcare expectations

Participants offered ways to decrease barriers to healthcare for those living with Long Covid symptoms, such as: changes to the primary care system including making it easier to get an appointment, more face‐to‐face appointments and longer appointment times that are empathetic to patient need. Participants wanted to feel listened to, unpressured by time constraints, have room to explore symptoms, reassurance that they are not wasting time, and to receive advice, treatment, signposting or referrals where appropriate from HCPs (Supporting Information S1: Material D, Quote 5).

Dedicated services for Long Covid were popular among participants, and these were in the form of helplines, webpages, specific appointments in GP practices for Long Covid and specialist clinics for people with Long Covid symptoms (Supporting Information S1: Material D, Quote 6). Some participants felt that access to these services should be available without visiting a GP.

Alternative forms of support were used when the NHS did not meet participants' needs and participants were open to social and community forms of support. Furthermore, there was a desire for change within primary care as well as dedicated Long Covid services.

## 4 DISCUSSION

To our knowledge, this study is the first to coproduce a community‐based approach to identify and include participants with probable Long Covid who may not be recognised as such by the health system. The coproduction process meant the inclusion criteria directly reflected the experiences of people living with Long Covid, which in turn meant that the study leaflet resonated with the intended audience. Subsequently, this led to engagement from people living with Long Covid symptoms that had previously not come forward for support.

It is evident from this analysis that a lack of adequate awareness of Long Covid, self‐doubt, hesitancy in seeking care and the nature of multisystem and changing symptoms act as barriers to seeking care for Long Covid symptoms. Participants' interviews also revealed experiences of discrimination, anticipated and internalised stigma which can further impede help‐seeking behaviour.

Some findings from this study show similarities with other Long Covid studies. Most notably, the varying range of symptoms experienced,^4^, ^5^, ^6^, ^7^, ^8^ the lack of Long Covid awareness among some population groups,^7^, ^47^, ^48^ self‐doubt regarding Long Covid as a cause for illness,^7^ symptoms as a barrier to accessing care,^47^ problems accessing primary care,^6^, ^10^, ^49^ experiences of symptoms being dismissed,^10^, ^32^, ^50^ seeking support outside the NHS and making behavioural changes,^6^, ^11^, ^49^, ^50^, ^51^ as well as stigma^6^, ^50^, ^51^ and discrimination^49^ which can all act as barriers to obtaining support and treatment.

**Figure 2:**
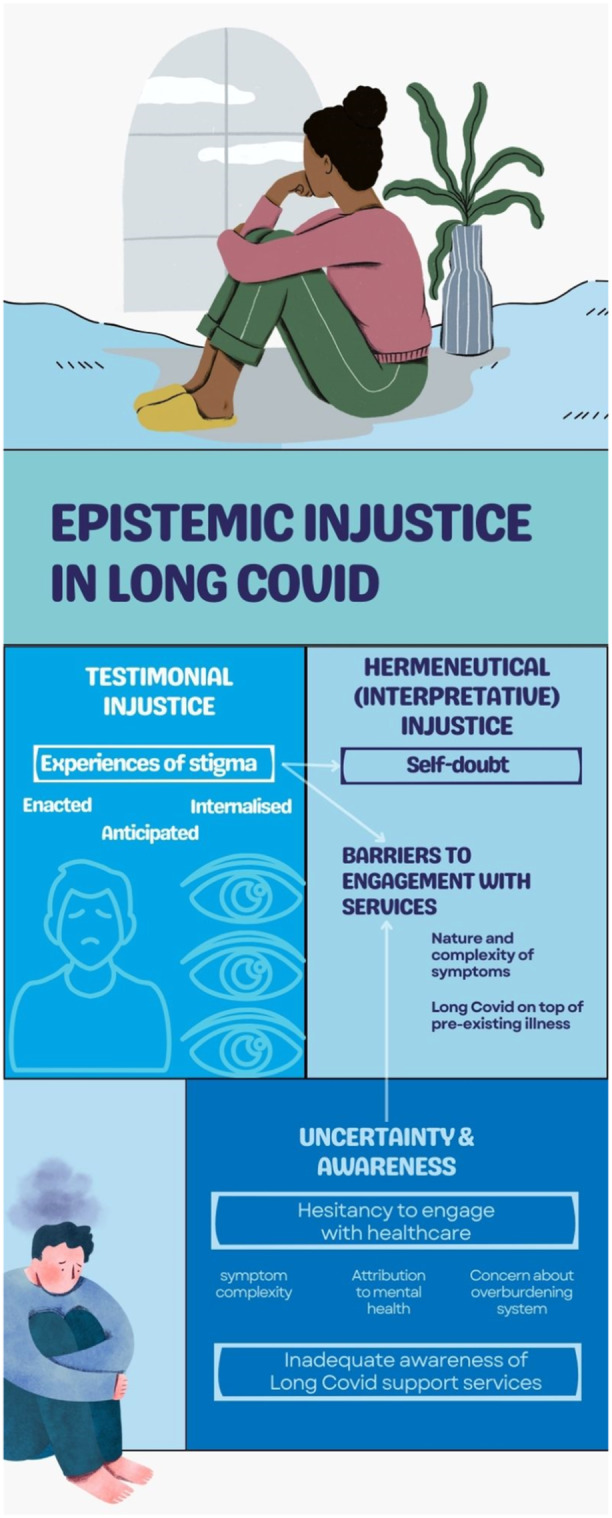
Epistemic injustice in Long Covid as a framework for the study findings. Graphic created using Canva software.

Parallels can also be drawn with Ireson et al.^32^ who suggest the experiences of people living with Long Covid, equate to epistemic injustice. As Bhakuni and Abimbola^52^ explain, testimonial injustice happens when a hearer prejudicially ascribes lower credibility to a speaker's words, for example, through acts that silence, undervalue, or distort. Hermeneutical (or Interpretive) injustice happens with someone struggles to make sense of and share their experience of the world, owing to a gap in available legitimised collective sensemaking resources. This particularly occurs when the experiences of marginalised individuals or groups are not understood by themselves or by others because those experiences do not fit any concepts known to them (or to others). Our findings fit under both testimonial and hermeneutical injustice frameworks (Figure 2).

There is inherently a power imbalance in the patient–HCP relationship. HCPs are unsurprisingly considered more medically knowledgeable, and they also have the power to decide suitable treatment options for different patients. These options are decided based on which patient narratives HCPs define as believable or unbelievable (testimonial injustice).^53^, ^54^ This can result in some patients being dismissed, as in this study, and in similar under‐recognised and under‐researched conditions such as chronic pain or myalgic encephalomyelitis.^54^, ^55^ On the other hand, patients may not have the ability to convey their symptoms in a way that is understandable by HCPs (hermeneutical injustice).^53^, ^54^ These experiences are compounded by inadequate awareness, knowledge and understanding of Long Covid, both within the community and at the healthcare level, due to its novel status, and also perhaps due to inadequate reference to Long Covid as a result of deficient public health messaging about the potential long‐term health effects of COVID‐19.

Experiences of both anticipated and internalised stigma were presented in this study. One explanation could be because individuals have internalised expectations of how they should be, and if they do not meet these expectations, they feel stigmatised.^27^ For people with Long Covid, this could partly be due to COVID‐19 being described as a ‘mild’, self‐limiting illness that most people recover from quickly—a definition which is evidently contested.^32^, ^50^, ^56^, ^57^ Those who experience ongoing symptoms from COVID‐19 are situated outside this dominant definition of ‘mildness’ which can result in feelings of being flawed.^27^

This study advances the health stigma literature by highlighting how stigma can lead to the discounting of the experiences of people with Long Covid due to negative stereotyping. When multiple forms of marginalisation are compounded by experiencing a stigmatised health condition, this results in layered or intersectional stigma,^58^, ^59^ which has been described in other health conditions too.

Experiences of stigma can prevent people living with Long Covid from seeking and/or receiving much‐needed support for their symptoms. They can lead to self‐doubt and hesitancy in seeking care. Self‐doubt surrounding the cause of, and finding other explanations for, symptoms can act as a barrier to obtaining care for chronic conditions.^29^ Bury explains this as a way of attempting to regain some ‘control’ as well as minimise the perceived damage to identities.^29^

Unlike in other studies,^6^, ^50^, ^51^ these participants did not identify online communities as a key source of support. This could be due to the lack of awareness of Long Covid in this group. Participants were receptive to potential forms of social and community support, nonetheless. Stigma can be minimised when people know that they are not alone in their experiences. Additionally, financial and economic support were considered useful, strengthening the argument that Long Covid, like other LTCs, can create social and economic burdens.^4^, ^9^ Future research should explore which forms of social and community support—both online and offline—are useful for different population groups living with Long Covid, with the particular aim of evaluating what works to reduce health inequalities.

**Figure 3:**
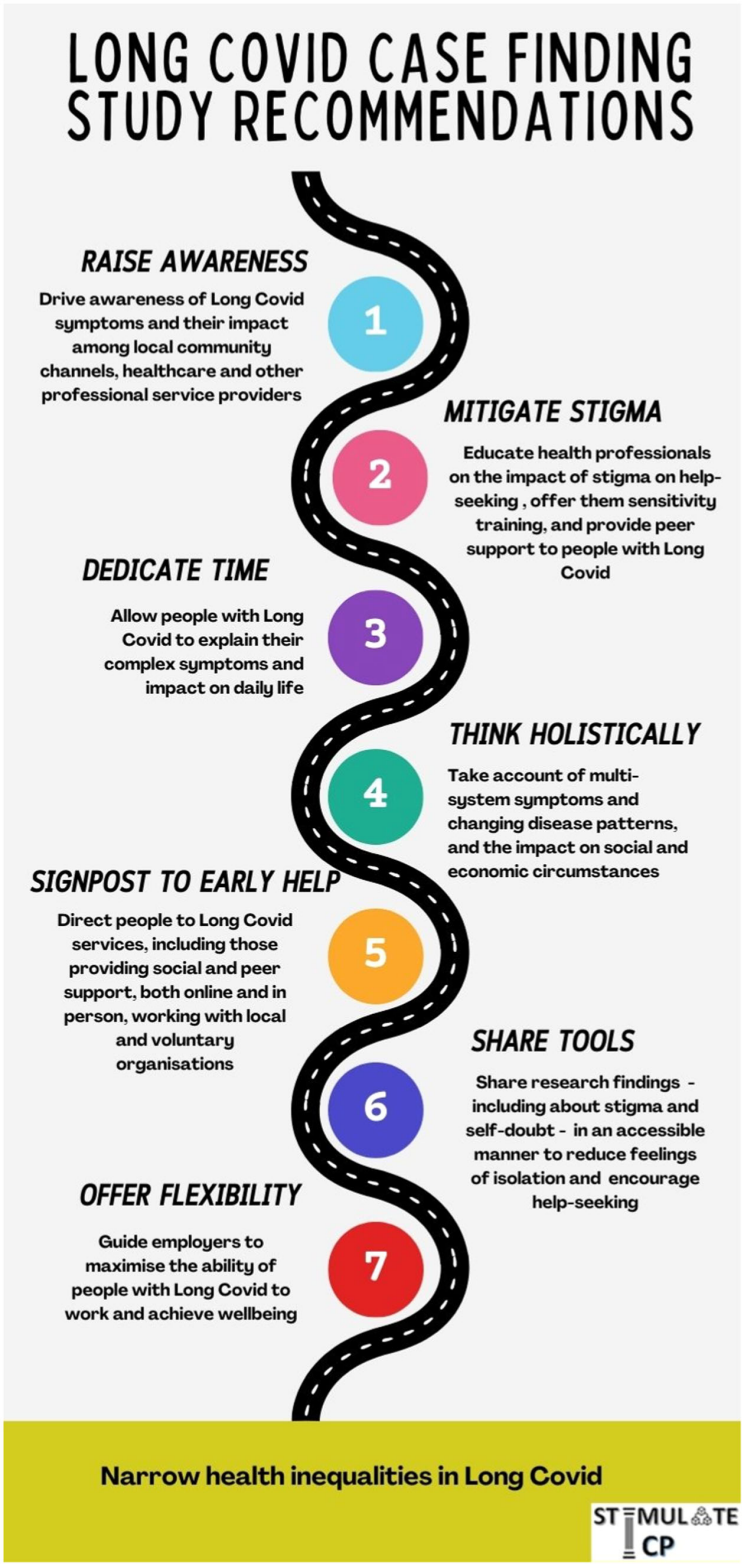
Long Covid active case finding study recommendations. Graphic created using Canva software.

The study recommendations are derived from the findings and are summarised in Figure 3. We are producing an online tool to facilitate help‐seeking in people with Long Covid who are struggling to access support. It is important to recognise that although all participants in this study reported symptoms consistent with Long Covid, alternative diagnoses are possible. Regardless of the diagnosis, people living with these symptoms must be encouraged to seek medical help and diagnosis.

The coproduced nature of this study is a strength. Including the voices of people with lived experience of Long Covid has resulted in research that is more relevant to others experiencing Long Covid symptoms. The case finding strategy used in this study offers one way to identify cases of probable Long Covid. Long Covid cannot be proven by one diagnostic test and is usually diagnosed based on symptoms. The eligibility and screening criteria used in this study are based on the WHO Clinical Case Definition of post‐COVID‐19 condition.^1^

In comparison with other Long Covid studies, our study also offers a different perspective of viewing the barriers that people with Long Covid face in accessing support through the lens of stigma and provides depth to the LCSS stigma dimensions. Despite efforts aimed at doing so, we were unable to recruit younger people (<29 years), so we do not know if the experiences for people with Long Covid symptoms in their late teens or twenties are the same as for older age groups. Future research should attempt to include the experiences of young adults.

We successfully employed a codesigned active case finding strategy to identify cases of probable Long Covid in three local communities in England. Our findings have highlighted the different, sometimes intersecting, barriers that can prevent people living with Long Covid from obtaining a diagnosis, treatment, or care for their symptoms. Despite Long Covid being a relatively new illness, it can be a stigmatising experience congruent with epistemic injustice. Action on raising awareness, mitigating stigma, listening to people with lived experience, and signposting to early help in the health and wider services are needed.

## AUTHOR CONTRIBUTIONS

**Donna Clutterbuck**: Investigation; writing—original draft; methodology; writing—review and editing; formal analysis; project administration. **Mel Ramasawmy**: Writing—original draft; writing—review and editing; formal analysis. **Marija Pantelic**: Conceptualisation; methodology; funding acquisition; writing—review and editing. **Jasmine Hayer**: Conceptualisation; funding acquisition; methodology; writing—review and editing. **Fauzia Begum**: Writing—review and editing; methodology. **Mark Faghy**: Writing—review and editing; methodology. **Nayab Nasir**: Writing—review and editing; methodology. **Barry Causer**: Writing—review and editing; methodology. **Melissa Heightman**: Methodology; writing—review and editing. **Gail Allsopp**: Conceptualisation; funding acquisition; methodology; writing—review and editing. **Dan Wootton**: Methodology; writing—review and editing. **M. Asad Khan**: Methodology; writing—review and editing. **Claire Hastie**: Writing—review and editing; methodology. **Monique Jackson**: Methodology; writing—review and editing. **Clare Rayner**: Methodology; writing—review and editing. **Darren Brown**: Writing—review and editing; methodology. **Emily Parrett**: Writing—review and editing; methodology. **Geraint Jones**: Writing—review and editing; methodology. **Rowan Clarke**: Writing—review and editing; methodology. **Sammie Mcfarland**: Writing—review and editing; methodology. **Mark Gabbay**: Writing—review and editing; conceptualisation; funding acquisition. **Amitava Banerjee**: Conceptualisation; funding acquisition; writing—review and editing. **Nisreen A. Alwan**: Conceptualisation; funding acquisition; writing—original draft; methodology; writing—review and editing; formal analysis; project administration.

## CONFLICT OF INTEREST STATEMENT

Clare Rayner is a member of the Society of Occupational Medicine's Long Covid Taskforce, has received occasional honoraria for talks given on Long Covid, has done occasional paid consultancy work for employers. Nisreen A. Alwan is a scientific advisor to the Long Covid Support Charity and has contributed in an advisory capacity to WHO and the EU Commission's Expert Panel on effective ways of investing in health meetings in relation to post‐COVID‐19 condition. Clare Rayner and Nisreen A. Alwan are Long Covid Kids Charity Champions. The other authors declare no conflict of interest.

## ETHICS STATEMENT

This study received ethical approval from the University of Southampton Faculty of Medicine Ethics Committee on 27 July 2022 (reference number 72400).

## Supporting information

Supplementary Information

## Data Availability

The anonymised data that support the findings of this study can be made available on reasonable request from the corresponding authors.

## References

[1] World Health Organization . A clinical case definition of post COVID‐19 condition by a Delphi consensus. 2023. Accessed July 12, 2023. https://www.who.int/publications/i/item/WHO-2019-nCoV-Post_COVID-19_condition-Clinical_case_definition-2021.1

[2] Nabavi N . Long covid: how to define it and how to manage it. BMJ. 2020;370:m3489. 10.1136/bmj.m3489 32895219

[3] Office for National Statistics . Prevalence of ongoing symptoms following coronavirus (COVID‐19) infection in the UK. March 30, 2023. Accessed August 10, 2023. https://www.ons.gov.uk/peoplepopulationandcommunity/healthandsocialcare/conditionsanddiseases/bulletins/prevalenceofongoingsymptomsfollowingcoronaviruscovid19infectionintheuk/30march2023

[4] Ziauddeen N , Gurdasani D , O'Hara ME , et al. Characteristics and impact of Long Covid: findings from an online survey. PLoS One. 2022;17(3):e0264331. 10.1371/journal.pone.0264331 35259179 PMC8903286

[5] Buttery S , Philip KEJ , Williams P , et al. Patient symptoms and experience following COVID‐19: results from a UK‐wide survey. BMJ Open Respir Res. 2021;8:e001075. 10.1136/bmjresp-2021-001075 PMC8572361 34732518

[6] Ladds E , Rushforth A , Wieringa S , et al. Persistent symptoms after Covid‐19: qualitative study of 114 “long Covid” patients and draft quality principles for services. BMC Health Serv Res. 2020;20(1):1144. 10.1186/s12913-020-06001-y 33342437 PMC7750006

[7] Cooper E , Lound A , Atchison CJ , et al. Awareness and perceptions of Long COVID among people in the REACT programme: early insights from a pilot interview study. PLoS One. 2023;18(1):e0280943. 10.1371/journal.pone.0280943 36701357 PMC9879384

[8] Woodrow M , Carey C , Ziauddeen N , et al. Systematic review of the prevalence of Long COVID. Open Forum Infect Dis. 2023;10(7):ofad233. 10.1093/ofid/ofad233 37404951 PMC10316694

[9] Health Talk . Family experiences of Long Covid: financial impact of Long Covid/household economics/resources. 2023. Accessed September 6, 2023. https://healthtalk.org/Family-experiences-of-Long-Covid/Financial-impact-of-Long-Covidhousehold-economicsresources

[10] Owen R , Ashton RE , Skipper L , et al. Long COVID quality of life and healthcare experiences in the UK: a mixed method online survey. Qual Life Res. 2024;33:133‐143. 10.1007/s11136-023-03513-y 37740144 PMC10784347

[11] Callum T , Mark AF , Rebecca O , et al. Lived experience of patients with Long COVID: a qualitative study in the UK. BMJ Open. 2023;13(4):e068481. 10.1136/bmjopen-2022-068481 PMC10151237 37185640

[12] The King's Fund . The health of people from ethnic minority groups in England. 2023. Accessed August 15, 2023. https://www.kingsfund.org.uk/publications/health-people-ethnic-minority-groups-england

[13] Jayasinghe S , Faghy MA , Hills AP . Social justice equity in healthy living medicine—an international perspective. Prog Cardiovasc Dis. 2022;71:64‐68. 10.1016/j.pcad.2022.04.008 35490871

[14] Public Health England . Health profile for England. 2021. Accessed August 15, 2023. https://fingertips.phe.org.uk/static-reports/health-profile-for-england/hpfe_report.html

[15] Office for National Statistics . Coronavirus (COVID‐19) case rates by socio‐demographic characteristics, England. September 1, 2020 to December 10, 2021. Accessed August 15, 2023. https://www.ons.gov.uk/peoplepopulationandcommunity/healthandsocialcare/conditionsanddiseases/bulletins/coronaviruscovid19caseratesbysociodemographiccharacteristicsengland/1september2020to10december2021

[16] Shabnam S , Razieh C , Dambha‐Miller H , et al. Socioeconomic inequalities of Long COVID: a retrospective population‐based cohort study in the United Kingdom. J R Soc Med. 2023;116(8):263‐273. 10.1177/01410768231168377 37164035 PMC10469969

[17] Jacobs MM , Evans E , Ellis C . Racial, ethnic, and sex disparities in the incidence and cognitive symptomology of long COVID‐19. J Natl Med Assoc. 2023;115(2):233‐243. 10.1016/j.jnma.2023.01.016 36792456 PMC9923441

[18] Chilunga FP , Appelman B , van Vugt M , et al. Differences in incidence, nature of symptoms, and duration of long COVID among hospitalised migrant and non‐migrant patients in the Netherlands: a retrospective cohort study. Lancet Reg Health Eur. 2023;29:100630. 10.1016/j.lanepe.2023.100630 37261215 PMC10079482

[19] Heightman M , Prashar J , Hillman TE , et al. Post‐COVID‐19 assessment in a specialist clinical service: a 12‐month, single‐centre, prospective study in 1325 individuals. BMJ Open Respir Res. 2021;8:e001041. 10.1136/bmjresp-2021-001041 PMC8587466 34764200

[20] Pantelic M , Ziauddeen N , Boyes M , O'Hara ME , Hastie C , Alwan NA . Long Covid stigma: estimating burden and validating scale in a UK‐based sample. PLoS One. 2022;17(11):e0277317. 36417364 10.1371/journal.pone.0277317 PMC9683629

[21] Kingstone T , Taylor AK , O'Donnell CA , Atherton H , Blane DN , Chew‐Graham CA . Finding the “right” GP: a qualitative study of the experiences of people with long‐COVID. BJGP Open. 2020;4:bjgpopen20X101143. 10.3399/bjgpopen20X101143 PMC7880173 33051223

[22] Scambler G , Hopkins A . Being epileptic: coming to terms with stigma. Sociol Health Illn. 1986;8:26‐43. 10.1111/1467-9566.ep11346455

[23] Markowitz FE . The effects of stigma on the psychological well‐being and life satisfaction of persons with mental illness. J Health Soc Behav. 1998;39:335‐347. 10.2307/2676342 9919855

[24] Earnshaw VA , Chaudoir SR . From conceptualizing to measuring HIV stigma: a review of HIV stigma mechanism measures. AIDS Behav. 2009;13(6):1160‐1177. 10.1007/s10461-009-9593-3 19636699 PMC4511707

[25] Pantelic M , Sprague L , Stangl AL . It's not “all in your head”: critical knowledge gaps on internalized HIV stigma and a call for integrating social and structural conceptualizations. BMC Infect Dis. 2019;19(1):210. 10.1186/s12879-019-3704-1 30832613 PMC6399894

[26] Goffman E . Stigma: Notes on the Management of Spoiled Identity. Touchstone; 2009.

[27] Scambler G . Health‐related stigma. Sociol Health Illn. 2009;31(3):441‐455. 19366430 10.1111/j.1467-9566.2009.01161.x

[28] Stangl AL , Earnshaw VA , Logie CH , et al. The health stigma and discrimination framework: a global, crosscutting framework to inform research, intervention development, and policy on health‐related stigmas. BMC Med. 2019;17(1):31. 10.1186/s12916-019-1271-3 30764826 PMC6376797

[29] Bury M . The sociology of chronic illness: a review of research and prospects. Sociol Health Illn. 1991;13(4):451‐468.

[30] Schnyder N , Panczak R , Groth N , Schultze‐Lutter F . Association between mental health‐related stigma and active help‐seeking: systematic review and meta‐analysis. Br J Psychiatry. 2017;210(4):261‐268. 10.1192/bjp.bp.116.189464 28153928

[31] Phelan SM , Burgess DJ , Yeazel MW , Hellerstedt WL , Griffin JM , van Ryn M . Impact of weight bias and stigma on quality of care and outcomes for patients with obesity. Obes Rev. 2015;16(4):319‐326. 10.1111/obr.1226 25752756 PMC4381543

[32] Ireson J , Taylor A , Richardson E , Greenfield B , Jones G . Exploring invisibility and epistemic injustice in Long Covid—a citizen science qualitative analysis of patient stories from an online Covid community. Health Expect. 2022;25:1753‐1765. 10.1111/hex.13518 35557480 PMC9327841

[33] Fricker M . Epistemic Injustice: Power and the Ethics of Knowing. Oxford University Press; 2007. 10.1093/acprof:oso/9780198237907.001.0001

[34] Alwan NA , Clutterbuck D , Pantelic M , et al. Long Covid active case finding study protocol: a co‐produced community‐based pilot within the STIMULATE‐ICP study (Symptoms, Trajectory, Inequalities and Management: Understanding Long‐COVID to Address and Transform Existing Integrated Care Pathways. PLoS One. 2023;18(7):e0284297. 10.1371/journal.pone.0284297 37471432 PMC10358953

[35] NIHR . Guidance on co‐producing a research project. April 2021. Accessed July 19, 2023. https://www.learningforinvolvement.org.uk/content/resource/nihr-guidance-on-co-producing-a-research-project

[36] Redman S , Greenhalgh T , Adedokun L , Staniszewska S , Denegri S , Co‐production of Knowledge Collection Steering C . Co‐production of knowledge: the future. BMJ. 2021;372(434):434. 10.1136/bmj.n434 PMC7884902 33593753

[37] Alwan NA . Lessons from Long COVID: working with patients to design better research. Nat Rev Immunol. 2022;22:201‐202. 10.1038/s41577-022-00692-6 35169259 PMC8853146

[38] Camden Council . Camden Profile. March 2023. Accessed July 27, 2023. https://opendata.camden.gov.uk/People-Places/Camden-Profile-latest-/9m7e-5qyt

[39] Merton Council . Merton health and wellbeing strategy 2019‐24: a healthy place for healthy lives. 2023. Accessed August 16, 2023. https://www.merton.gov.uk/system/files?file=health20and20wellbeing20strategy20201920final20web.pdf

[40] Derbyshire County Council . Equality, diversity and inclusion strategy 2022–2025. 2023. Accessed August 16, 2023. https://www.derbyshire.gov.uk/site-elements/documents/pdf/council/equalities/equality-diversity-and-inclusion-strategy-2022-to-2025.pdf

[41] Merton Council . Population report for Merton. 2023. Accessed September 6, 2023. https://data.merton.gov.uk/population/#/view-report/63aeddf1d7fc44b8b4dffcd868e84eac/___iaFirstFeature/G3

[42] Derbyshire County Council . Ethnic group, language and religion. November 30, 2022. Accessed August 31, 2023. https://storymaps.arcgis.com/stories/7090e2e51bdf46648ee48186850ff201

[43] The King's Fund . What is social prescribing? 2023. Accessed September 26, 2023. https://www.kingsfund.org.uk/publications/social-prescribing

[44] Braun V , Clarke V . Thematic analysis. In: Cooper H , Camic PM , Long DL , Panter AT , Rindskopf D , Sher KJ , eds. APA Handbook of Research Methods in Psychology. Vol 2. American Psychological Association; 2012:57‐71.

[45] Braun V , Clarke V . Using thematic analysis in psychology. Qual Res Psychol. 2006;3:77‐101.

[46] Lumivero . NVivo (Version 13, R1). 2020.

[47] Kaufmann J , Gould O , Lloyd V . Seeking care for Long COVID: a narrative analysis of Canadian experiences. J Patient Exp. 2023;10:237437352311517. 10.1177/23743735231151770 PMC9880455 36710996

[48] Ipsos . Long COVID patient experience research summary report—Strands 1, 2, 3 and 4. March 2023. Accessed August 3, 2023. https://pexlib.net/?240309

[49] Baz SA , Fang C , Carpentieri JD , Sheard L . ‘I don't know what to do or where to go’. Experiences of accessing healthcare support from the perspectives of people living with Long Covid and healthcare professionals: a qualitative study in Bradford, UK. Health Expect. 2023;26:542‐554. 10.1111/hex.13687 36512382 PMC10124541

[50] Rushforth A , Ladds E , Wieringa S , Taylor S , Husain L , Greenhalgh T . Long Covid—the illness narratives. Soc Sci Med. 2021;286:114326. 10.1016/j.socscimed.2021.114326 34425522 PMC7617381

[51] Macpherson K , Cooper K , Harbour J , Mahal D , Miller C , Nairn M . Experiences of living with long COVID and of accessing healthcare services: a qualitative systematic review. BMJ Open. 2022;12:e050979. 10.1136/bmjopen-2021-050979 PMC8753091 35017239

[52] Bhakuni H , Abimbola S . Epistemic injustice in academic global health. Lancet Glob Health. 2021;9(10):e1465‐e1470. 10.1016/S2214-109X(21)00301-6 34384536

[53] Carel H , Kidd IJ . Epistemic injustice in healthcare: a philosophial analysis. Med Health Care Philos. 2014;17(4):529‐540. 10.1007/s11019-014-9560-2 24740808

[54] Buchman DZ , Ho A , Goldberg DS . Investigating trust, expertise, and epistemic injustice in chronic pain. J Bioeth Inq. 2017;14(1):31‐42. 10.1007/s11673-016-9761-x 28005251

[55] Blease C , Carel H , Geraghty K . Epistemic injustice in healthcare encounters: evidence from chronic fatigue syndrome. J Med Ethics. 2017;43(8):549‐557. 10.1136/medethics-2016-103691 27920164

[56] Alwan NA . What exactly is mild covid‐19? BMJ Opin. 2020. Accessed August 14, 2023. https://blogs.bmj.com/bmj/2020/07/28/nisreen-a-alwan-what-exactly-is-mild-covid-19/

[57] Callard F . Very, very mild: Covid‐19 symptoms and illness classification. Somatosphere. 2020.Accessed August 14, 2023. https://somatosphere.com/2020/mild-covid.html/?format=pdf

[58] MacLean L , Edwards N , Garrard M , Sims‐Jones N , Clinton K , Ashley L . Obesity, stigma and public health planning. Health Promot Int. 2008;24(1):88‐93. 10.1093/heapro/dan041 19131400

[59] Amon JJ , Sun N , Iovita A , Jurgens R , Csete J . Addressing stigma is not enough. Health Hum Rights. 2022;24(2):111‐114. 36579301 PMC9790942

