# Supplementary material for: Barriers to healthcare access and experiences of stigma: Findings from a coproduced Long Covid case‐finding study
Source: Health Expect. 2024 Apr 18;27(2):e14037. doi: 10.1111/hex.14037 (PMC11024953; doi:10.1111/hex.14037)
Supplement: Supplementary file 1 — Supplementary Information [file HEX-27-e14037-s001.docx]

**Supplementary materials**

Supplementary material A: Eligibility Criteria

Potential participants who engaged with the leaflet were encouraged to contact the research team to check eligibility. Study eligibility was based on confirmation of:

1. History of probable or confirmed COVID-19 infection at least three months before the time of recruitment
2. Prolonged symptoms* after COVID-19 lasting at least 2 months that cannot be explained by another condition
3. Symptoms’ impact on everyday functioning
4. No existing clinical diagnosis of Long Covid or uncertainty regarding diagnosis.

*Participants were included if they had three or more of the symptoms listed below (based on the evidence which points to Long Covid as predominantly a multi-symptom and multisystem condition as well as discussions with the CAB):

Post-exertional symptom exacerbation (symptoms such as fatigue, difficulty thinking, pain recurring after remission or getting worse following exertion, either immediately or up to 72 hours post exertion), exhaustion, cognitive dysfunction (brain fog, memory problems, concentration problems, occasional confusion), breathlessness, headache, muscle aches, palpitations, chest tightness/pressure, chest pain, dizziness, sleep disturbance, joint pain, leg pain, pins and needles feeling, tinnitus, sore throat, cough, nasal symptoms, hoarse voice, abdominal pain, nausea, diarrhoea, chills, altered or loss of sense of smell, altered or loss of sense of taste, skin rash, loss of appetite and sneezing.

Those who fulfilled the eligibility criteria above were invited to take part in the study.


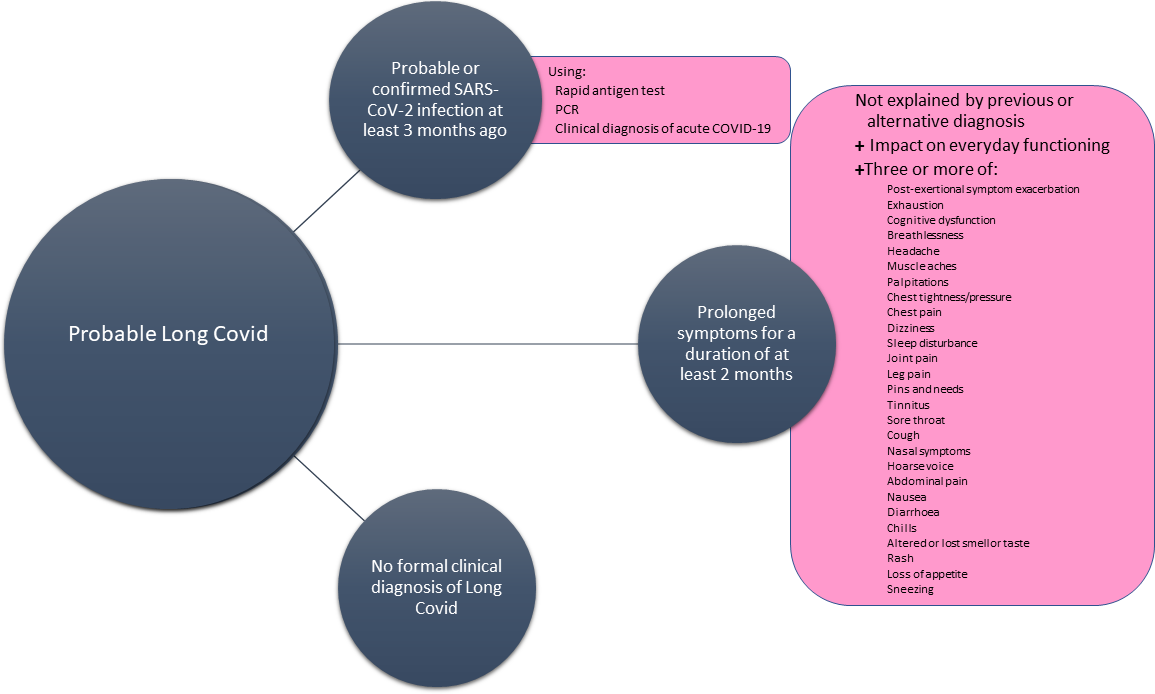
Image source: Alwan NA, Clutterbuck D, Pantelic M, Hayer J, Fisher L, Hishmeh L, et al. (2023) Long Covid active case finding study protocol: A co-produced community-based pilot within the STIMULATE-ICP study (Symptoms, Trajectory, Inequalities and Management: Understanding Long-COVID to Address and Transform Existing Integrated Care Pathways). PLoS ONE 18(7): e0284297. <https://doi.org/10.1371/journal.pone.0284297>

Supplementary material B: Screening Questions

**(To be administered by the researcher)**

**Researcher script (verbal consent to ask questions):**

*Thank you for contacting us about this study. Please can I ask where you heard about us?

………………………………………………………………………[Insert where the participant heard about the study here].*

*In order for us to check your eligibility for the study, please can I check that it is OK for us to ask you a few questions?*

Yes 🞏 No 🞏 - *If yes, continue to ask screening questions.*

1. a) Do you think you had Covid 3+ months ago?
    Yes 🞏 No 🞏 - *If yes continue to b)*

b) Was this suspected or confirmed Covid?
 Suspected 🞏 Confirmed 🞏 *If confirmed continue to c), suspected skip to 2)*

c) How was Covid confirmed?
 PCR test 🞏 Rapid antigen test (lateral flow) 🞏 Antibody test 🞏

1. Have you experienced prolonged symptoms after Covid infection?
   Yes 🞏 No 🞏 *– If yes, continue to question 3*
2. Do these symptoms restrict or limit your usual daily activities in any way (the usual activities you used to do before including work patterns, exercise and social activities)?

Yes 🞏 No 🞏 *– If yes, continue to question 4*

1. Have you been told by your GP that you have Long Covid?
   Yes 🞏 No 🞏 - *if yes, participant is not eligible to take part in the study*
2. a) Have you experienced any three of these symptoms for a duration of at least 2 months that are not explained by an existing health condition you already have – *If three or more are ticked from a) and b), participant is eligible to take part.*

- Exhaustion
- Brain fog
- Memory problems
- Concentration problems
- Occasional confusion
- Breathlessness
- Headache
- Muscle aches
- Palpitations
- Chest tightness/pressure
- Chest pain
- Dizziness
- Sleep disturbance
- Joint pain
- Leg pain
- Pins and needles feeling
- Tinnitus
- Sore throat
- Cough
- Nasal symptoms
- Hoarse voice
- Abdominal pain
- Nausea
- Diarrhoea
- Chills
- Altered or loss of sense of smell
- Altered or loss of sense of taste
- Skin rash
- Loss of appetite
- Sneezing

1. Do any of the symptoms described reoccur or get worse immediately or up to 72 hours following an increase in activity (PESE)?
   Yes 🞏 No 🞏

Those who fulfil the screening criteria above are assumed to have probable Long Covid and can be asked if they are interested in taking part in the study.

Supplementary material C: Symptoms

Participants suffered a range of symptoms consistent with Long Covid including exhaustion, brain fog, memory problems, concentration problems, occasional confusion, breathlessness, muscle aches, chest tightness/pressure, headache, dizziness, sleep disturbance, leg pain, tinnitus, cough, nasal symptoms, hoarse voice, nausea, diarrhoea, loss of appetite, sneezing, food allergies, altered sensations in the body, palpitations, skin rash and altered or lost sense of smell or taste. Some participants saw an exacerbation of existing conditions.

Participants reported experiencing symptoms for varying time periods, with some suffering from the acute COVID-19 infection in early 2020 and others in early 2023. Some had multiple infections. Most participants had this confirmed by polymerase chain reaction (PCR) or rapid lateral flow (LFT) tests, though five participants had suspected infections prior to the widespread availability of testing. Most participants were not hospitalised during the acute phase of their COVID-19 illness.

Supplementary material D: Sources of Support

| Sources of support subtheme |  | Example Quote |
| --- | --- | --- |
| Sources of treatment outside of the NHS | 1 | *’It was my last port of call, I said, “I’m desperate to get this, if I have it privately it’ll cost me a lot.” “What is it?” he said, and then he looked it up and he said, “I have heard about it, people have talked to me about it, but I’m not sure if it will work. It may be one of those solutions that are a bit cranky but I’ll let you try.” And he prescribed it’.* (female, 70-79, White British) |
| Social and community support | 2 | *‘It’s a bit like anything isn’t it. If you’ve experienced something yourself then it’s nice to know that it’s not just you that it’s happening to and that you’ve got someone to relate to’.* (female, 50-59, White) |
|  | 3 | ‘A lot of people are saying they’ve got the same thing’ (female, 70-79, Jewish) |
|  | 4 | *‘My sisters are younger than me, they’re so young and they’re having the same problem.* (female, 30-39, Bangladeshi-British) |
| Desired healthcare expectations | 5 | *‘You would want to feel that they had the time to consider what you’re saying and what our symptoms are and not feel pressurised by lack of time’.* (female, 50-59, White British) |
|  | 6 | *‘If there was a body that you could go to where it would be, you’d be asked questions specifically about your experience with COVID and your experiences now and your symptoms now. Perhaps if there was a dedicated place you could go to for that, that would make it a lot, because then you’d be, people would be specifying on that subject rather than thinking oh it could be HRT or it could be this or could be that. At least they’d be asking you about that specifically at first’.* (female, 60-69, White British) |
